# Deletion of *pgi* gene in *E. coli* increases tolerance to furfural and 5-hydroxymethyl furfural in media containing glucose–xylose mixture

**DOI:** 10.1186/s12934-020-01414-0

**Published:** 2020-07-28

**Authors:** Syed Bilal Jilani, Chandra Dev, Danish Eqbal, Kamran Jawed, Rajendra Prasad, Syed Shams Yazdani

**Affiliations:** 1 Microbial Engineering Group, International Centre for Genetic Engineering and Biotechnology (ICGEB), Aruna Asaf Ali Marg, New Delhi, India; 2 DBT-ICGEB Centre for Advanced Bioenergy Research, International Centre for Genetic Engineering and Biotechnology, New Delhi, India; 3 Institute of Biotechnology, Amity University, Manesar, Haryana India; 4 Present Address: Biodiscovery Institute, School of Life Sciences, University of Nottingham, Nottingham, UK

**Keywords:** *Escherichia coli*, Furfural, 5-hydroxymethyl furfural, Glucose, Xylose, Co-utilization, Ethanol, Lignocellulose, Ethanol productivity

## Abstract

**Background:**

Furfural and 5-hydroxymethyl furfural (5-HMF) are key furan inhibitors that are generated due to breakdown of lignocellulosic sugars at high temperature and acidic treatment conditions. Both furfural and 5-HMF act in a synergistic manner to inhibit microbial metabolism and resistance to both is a desirable characteristic for efficient conversion of lignocellulosic carbon to ethanol. Genetic manipulations targeted toward increasing cellular NADPH pools have successfully imparted tolerance against furfural and 5-HMF. In present study, deletion of *pgi* gene as a strategy to augment carbon flow through pentose phosphate pathway (PPP) was studied in ethanologenic *Escherichia coli* strain SSK101 to impart tolerance towards either furfural or 5-HMFor both inhibitors together.

**Results:**

A key gene of EMP pathway, *pgi*, was deleted in an ethanologenic *E. coli* strain SSK42 to yield strain SSK101. In presence of 1 g/L furfural in minimal AM1 media, the rate of biomass formation for strain SSK101 was up to 1.9-fold higher as compared to parent SSK42 strain, and it was able to clear furfural in half the time. Tolerance to inhibitor was associated with glucose as carbon source and not xylose, and the tolerance advantage of SSK101 was neutralized in LB media. Bioreactor studies were performed under binary stress of furfural and 5-HMF (1 g/L each) and different glucose concentrations in a glucose–xylose mixture with final sugar concentration of 5.5%, mimicking major components of dilute acid treated biomass hydrolysate. In the mixture having 6 g/L and 12 g/L glucose, SSK101 strain produced ~ 18 g/L and 20 g/L ethanol, respectively. Interestingly, the maximum ethanol productivity was better at lower glucose load with 0.46 g/(L.h) between 96 and 120 h, as compared to higher glucose load where it was 0.33 g/(L.h) between 144 and 168 h. Importantly, parent strain SSK42 did not exhibit significant metabolic activity under similar conditions of inhibitor load and sugar concentration.

**Conclusions:**

*E. coli* strain *SSK101* with *pgi* deletion had enhanced tolerance against both furfural and 5-HMF, which was associated with presence of glucose in media. Strain SSK101 also had improved fermentation characteristics under both hyperosmotic as well as binary stress of furfural and 5-HMF in media containing glucose–xylose mixture.

## Background

Rapid industrialization of previous agriculture-based economies have placed unprecedented stress on sustainability of present-day energy requirements, which are primarily fossil fuel based. Lignocellulosic biomass presents a promising source of cellulosic carbon which can be channeled towards synthesis of compounds having potential in bioenergy industry. Major hindrance to economical extraction of bioenergy compounds from cellulosic carbon is the recalcitrance of lignocellulose to microbial activity [1]. Pretreatment of biomass is performed for efficient extraction of both hexose and pentose sugars from lignocellulose and to make it amenable to microbial metabolism. Among other methods, higher temperature and dilute acid treatment is frequently employed to depolymerize lignocellulose for efficient extraction of pentose sugars in a liquid concoction, which is termed as liquid hydrolysate [2, 3].

Hydrolysate represents a stressful environment due to presence of various inhibitors and mounts multidimensional challenge to microbial cell [4]. The inhibitors are a complex mix of minerals, organic acids, furans and phenolics, and concentration of each inhibitor varies depending upon the lignocellulosic source and treatment conditions. For example, in woody substrate a wide variation of acetic acid (1.8–11.5 g/L), furfural (0.2–4.6 g/L) and 5-HMF (0.2–8.6 g/L) has been reported [5]. On the other hand, in rice straw hydrolysate, relatively less variation in acetic acid (1.4–2.4 g/L), furfural (0.4–1.6 g/L) and 5-HMF (0.2–1.5 g/L) has been observed [6]. A relatively higher concentration of acetic acid (5.3–12.5 g/L) as compared to that of furfural (1.02–0.58 g/L), 5-HMF (0.28–0.75 g/L) and total soluble phenolics (0.70–2.86 g/L) has been reported from bagasse pretreatment [7, 8]. Furfural is a key aldehyde inhibitor which acts synergistically with other inhibitors and presents one of the most potent toxic combination with 5-HMF [9]. Different cellular components involved in maintaining physiological homeostasis have been involved in conferring tolerance against furfural [10, 11]. Components of DNA repair [10, 12], cell membrane and chaperones [11], and MDR efflux pumps [13] have been reported to confer tolerance against furfural. Causal mechanisms for toxicity of furfural to microbial cell have been suggested to be altered redox ratios [14], accumulation of ROS [15] and reduced biosynthesis of sulfur containing macromolecules [16]. Tolerance to furfural in *E. coli* has been conferred by deletion of NADPH utilizing genes *yqhD* and *dkgA* [17], overexpression of *fucO* [18, 19] which utilizes NADH as a cofactor, and overexpression of membrane bound transhydrogenase *pntAB* [16]. Deletion of *yqhD* and *dkgA* have also been attributed to increased tolerance against 5-HMF [20]. Thus, published literature points to the fact that modulation of the cellular redox machinery is a successful strategy to confer tolerance against both furfural and 5-HMF.

**Fig. 1:**
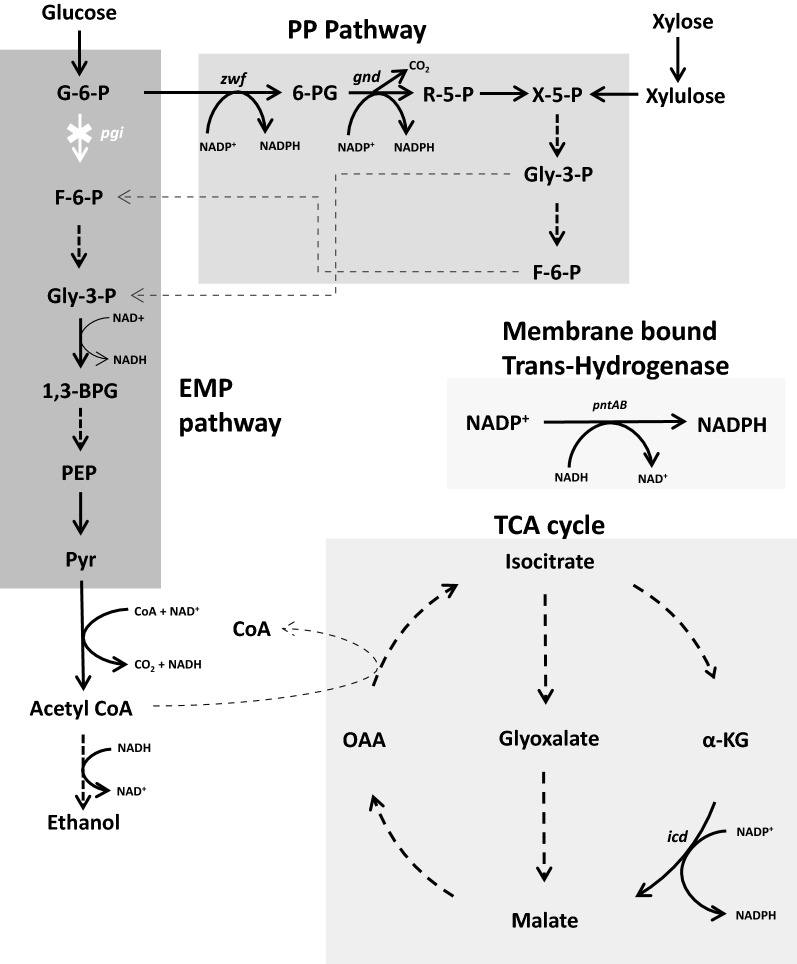
Flow of glucose and xylose carbon via central metabolic pathways in *Escherichia coli*. NADPH is generated via pentose phosphate (PP) pathway, tricarboxyic acid (TCA) cycle and membrane bound trans-hydrogenase. Xylose enters PP pathway at X5P step thus bypassing the NADP^+^ reducing steps catalyzed by glucose-6-phosphate dehydrogenase and 6-phosphogluconate dehydrogenase which are encoded by *zwf* and *gnd*, respectively. In *Δpgi* strain, pyr is generated via channeling of Gly-3-P and F-6-P from PP to Embden-Meyerhof-Paranas (EMP) pathway. Connecting intermediates are indicated with thin broken lines. Abbreviations are as follow: G-6-P, glucose-6-phosphate; X-5-P, xylulose-5-phosphate; gly-3-P, glyceraldehyde-3-phosphate; F-6-P, fructose-6-phosphate; PEP, phosphoenolpyruvate; Pyr, pyruvate; OAA, oxaloacetic acid

NADPH is the anabolic currency of the microbial cell. Three main sources for NADPH generation are (i) the oxidative phase of pentose phosphate (PP) pathway (ii) isocitrate dehydrogenase (Icd) of the Tricarboxylic acid (TCA) cycle and (iii) membrane bound PntAB transhydrogenase (Fig. 1). Deletion of the *pgi* gene of Embden-Meyerhof-Paranas (EMP) pathway shunts all glucose carbon via PP pathway and doubles up the production of NADPH via PP and Icd as compared to its utilization in *E. coli* [21]. The cellular machinery responds by dramatically reducing cellular growth rate [21, 22] and resumes growth only when a NADPH sink is introduced [23]. An evolutionary adaptation study of *pgi* mutant in glucose minimal media has reported mutants with either truncated *pntAB* or mutations attributed to upregulation of *udhA* which lead to relatively higher growth rates of all evolved strains as compared to the un-evolved parent [24]. *udhA* is a soluble transhydrogenase and reduces NAD^+^ using NADPH as a reductant. These studies suggest that the *E. coli* cell responds to excess NADPH by ceasing growth and resumes it only when either a NADPH sink or mutation in proteins involved in maintaining redox homeostasis is introduced.

By modulating endogenous pathway, we earlier reported engineering of an ethanol producing *E. coli* strain SSY09(pZSack) [25]. This strain was further engineered and evolved to increase ethanol productivity and titer to yield strain SSK42 [26]. In present study we knocked out *pgi* gene in SSK42 to generate strain SSK101. We hypothesized that with glucose as carbon source SSK101 will have higher tolerance against both furfural and 5-HMF as compared to the parent strain SSK42 having functional *pgi* gene. Our results support the hypothesis and demonstrate three key findings. *Firstly*, presence of furfural confers growth advantage to SSK101 as compared to parent strain SSK42. *Secondly*, furfural is relatively more inhibitory to microbial metabolism as compared to 5-HMF. *Finally*, SSK101 has growth advantage as compared to SSK42 under combined hyperosmotic and binary stress of furfural and 5-HMF.

## Results and discussion

### Impact of furfural and 5-HMF on rate of biomass production

Earlier studies [16, 17] suggest that in presence of furfural, microbial cell shunts NADPH away from anabolic reactions towards detoxification of furfural which leads to decrease in biomass yields. Meanwhile, deletion of *pgi* gene shunts all glucose carbon via PPP and leads to excess production of NADPH [21]. The excess NADPH itself acts as an inhibitor and leads to growth arrest, which resumes only upon introduction of a NADPH sink [23]. We thus tested the hypothesis whether furfural and 5-HMF has potential to improve biomass formation rate in the *pgi* deleted *E. coli* strain SSK101. This proof of concept experiment was performed in shake flasks with AM1 medium that is a low salt mineral medium with an external carbon source [27].

**Fig. 2:**
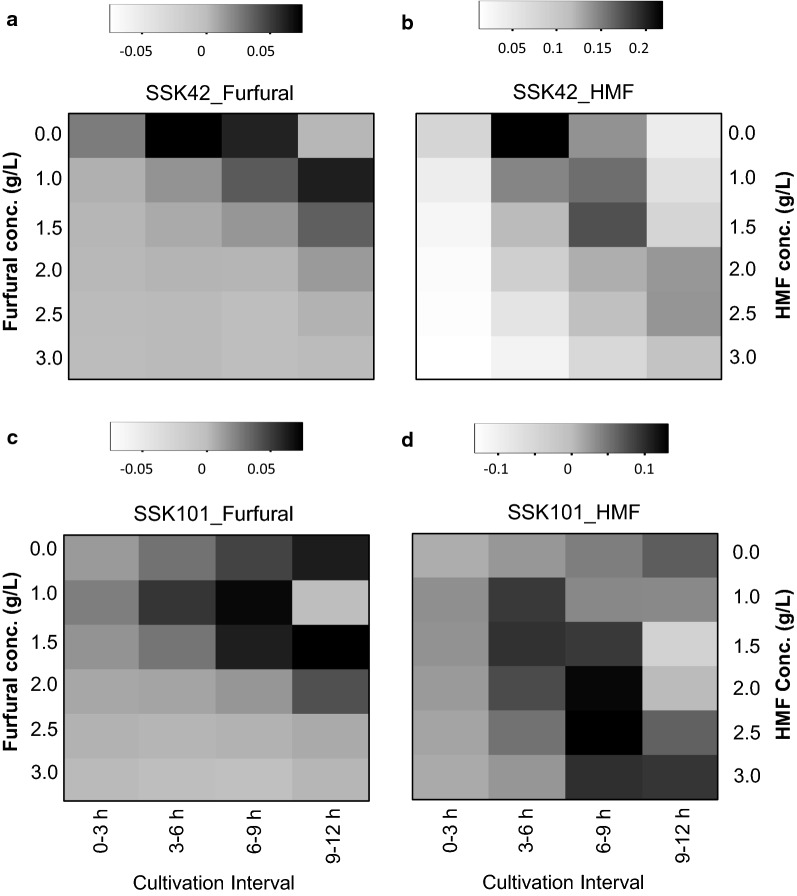
Comparison of rate of change in biomass of SSK42 and SSK101 in presence of inhibitor. Strain SSK42 (**a**, **b**) and SSK101 (**c**, **d**) were cultured in shake flasks in AM1 media (1% glucose) in presence of either furfural (**a**, **c**) or 5-HMF (**b**, **d**). Y-axis represents concentration of respective inhibitor (g/L) and X-axis the 3 h sampling interval. Rate of change in biomass in g/(L.h) was calculated for respective 3 h sampling window. Intensity of shading in each box represents extent of growth rate and has been normalized within each condition as mentioned in the Method section. Range of growth rates in g/(L.h) and its relation with intensity of shading has been indicated above each panel

When cells were grown in absence of furfural (0 g/L), the parent SSK42 strain achieved highest rate of biomass formation during 3–6 h cultivation interval (Fig. 2a) as compared to *pgi* deleted SSK101 strain that achieved highest value at 9–12 h interval of cultivation (Fig. 2c). Interestingly, when the growth rates of these two strains were monitored in presence of 1 to 3 g/L furfural, the trend was found to be reversed. With increasing concentration of furfural, SSK101 had relatively higher growth rate as compared to its parent strain SSK42 at corresponding cultivation interval (Fig. 2a, c). At 1.0 g/L furfural, the rate of change of biomass for SSK42 at 0–3 and 3–6 h intervals were 0.013 ± 0.004 g/(L.h)and 0.044 ± 0.005 g/(L.h), respectively (Additional file 1: Table S1), while the values for SSK101at corresponding sampling time were 0.025 ± 0.004 g/(L.h) (1.92-fold higher) and 0.054 ± 0.003 g/(L.h) (1.27-fold higher), respectively. Similar growth advantage for SSK101 was also observed at 1.5 g/L and 2 g/L furfural. These results suggested that presence of 1–2 g/L furfural in media promoted formation of biomass in SSK101. At 2.5 and 3.0 g/L furfural concentration no noticeable biomass formation rate was observed in either strain.

Along similar line, we investigated the influence of 5-HMF on the rate of change in biomass. In both SSK42 and SSK101, evidence of microbial growth was observed even at the highest tested concentration (3 g/L) of 5-HMF (Fig. 2b, d), suggesting that 5-HMF is relatively less inhibitory to microbial growth as compared to furfural. Similar to the pattern observed in case of furfural, presence of 5-HMF in media reduced the rate of biomass formation in SSK42, while it promoted the rate of biomass formation in SSK101 as compared to control where no 5-HMF was present in the growth media. However, though 5-HMF promoted biomass formation rate in strain SSK101, deletion of *pgi* gene didn’t result in any significant growth advantage to SSK101 as compared to the parent strain SSK42 in presence of 5-HMF (Additional file 1: Table S2).

The above results indicate that furfural and 5-HMF lead to an improvement in the biomass formation rate in the *pgi* deleted *E. coli* strain SSK101. This observation further compelled us to investigate the growth behavior of strain SSK101 over a finer range of furfural or 5-HMF. We hypothesized that there will be an optimum inhibitor concentration at which the value of rate of change of biomass will be maximum, and any inhibitor concentration lower or higher than that optimum concentration will result in lower values of biomass formation rate.

**Fig. 3:**
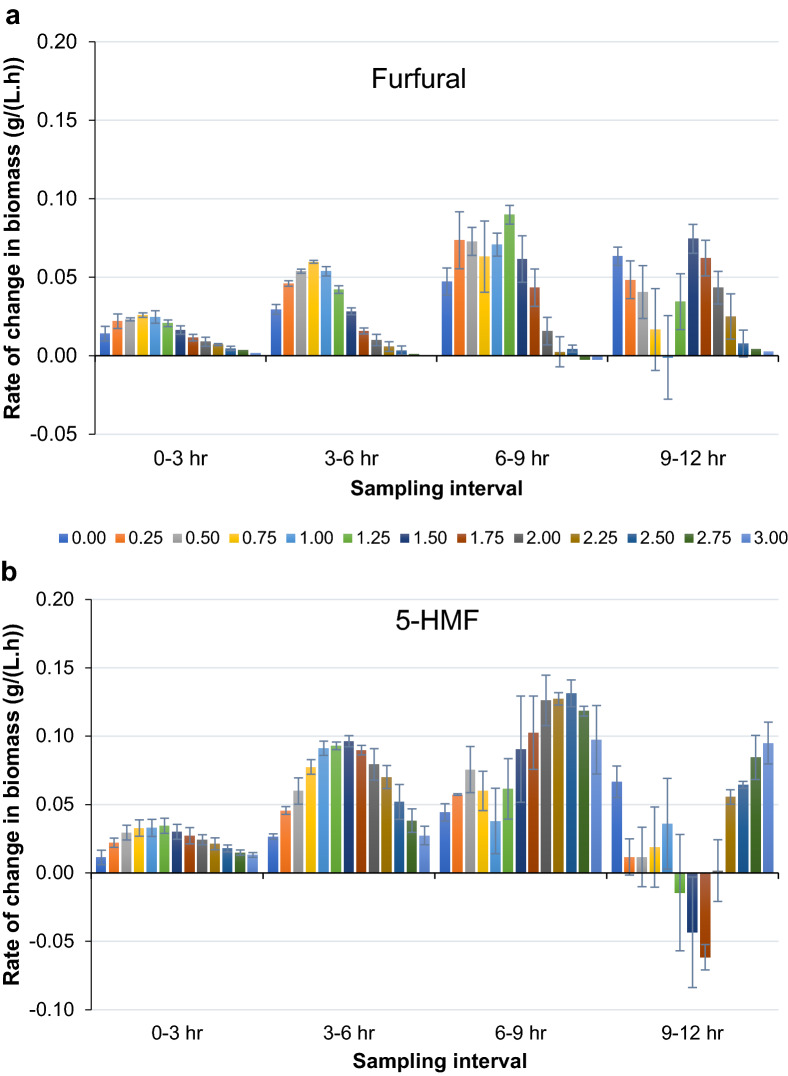
Detailed profile of rate of change in biomass of SSK101 in presence of different concentrations of inhibitors. Strain SSK101 was cultured in shake flasks in AM1 media (1% glucose) in presence of 0 to 3 g/L of either furfural (**a**) or 5-HMF (**b**). X-axis represents the sampling interval and Y-axis the rate of change in biomass (g/(L.h)). Sampling was performed at 3 h time interval

We observed a pattern in growth rate whereas compared to 0 g/L furfural, presence of furfural in the media until 1.50 g/L promoted rate of increase in biomass of strain SSK101 (Fig. 3a). Maximum value (p value = 0.003) was exhibited at 1.25 g/L during 6–9 h interval. Growth could still be observed till about 2.25 g/L but at a lower rate as compared to the control. At furfural concentration higher than 2.25 g/L the cellular metabolism is overwhelmed and no measurable change in biomass could be observed. Relative to furfural, SSK101 displayed higher growth rates at nearly all corresponding time points when 5-HMF was present in the medium (Fig. 3b). Presence of 5-HMF in media promoted rate of increase in biomass and reached maximum value (p-value = 0.001) at around 2.5 g/L during 6-9 h interval. Though increase in rate of change in biomass could be observed till 3.0 g/L as compared to 0 g/L 5-HMF, the values were lower than those at 2.5 g/L. Interestingly, we observed that with increase in time, the value of rate of change in biomass shifted to higher side at the higher respective inhibitor concentration, with concomitant decrease in values at lower inhibitor concentration. This observation suggests that as the inhibitor is cleared from the medium the strain loses its ability to increase biomass.

Based upon the results described above we predict that presence of either furfural or 5-HMF in the media promotes growth till the point where intracellular production of excess NADPH is balanced by the concentration of inhibitor. Any further decrease or increase in the inhibitor concentration leads to either NADPH glut or scarcity, respectively, which affects microbial anabolic reactions. We thus conclude that SSK101 exhibits the potential to act as a catalyst to remove furfural and 5-HMF from the media.

### Role of media components on furfural metabolism

From shake flask studies it became clear that both furfural and 5-HMF promote rate of biomass formation in strain SSK101 and not in parent strain SSK42. The data also suggests that furfural is relatively more inhibitory to microbial metabolism as compared to 5-HMF. To increase stringency in our further investigations, we chose furfural to investigate physiological responses of strains SSK101 and SSK42 in capped Hungate tube experiments. Closed tube experiments add second degree of stringency by limiting gaseous exchange with progress of microbial growth. In addition to tweaking with aerobicity, we also investigated the role of (i) different carbon sources, (ii) minimal and complex media, and (iii) osmotic stress, on microbial metabolism in presence of furfural.

#### Effect of different carbon source

**Fig. 4:**
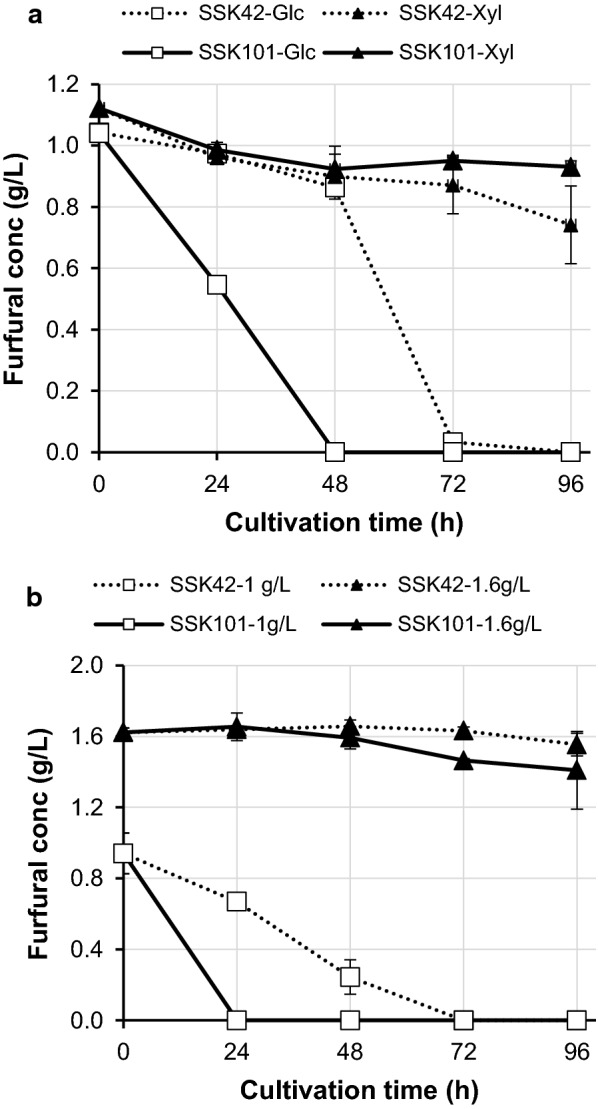
Influence of carbon source and osmotic stress on furfural metabolism in AM1 media. **a** SSK42 and SSK101 strains were grown in Hungate tubes in presence of furfural (1 g/L) and either 1% glucose or 1% xylose. **b** Strains were cultured in Hungate tubes in AM1 media containing a mixture of glucose (1%) and xylose (4%). Sampling was performed as indicated in the text

In case of SSK101 and glucose as carbon source, no furfural could be detected in media at 48 h, while SSK42 took additional 24 h to remove the inhibitor from medium (Fig. 4a). It is important to note that SSK42 can metabolize glucose via both EMP and PP pathways, which would result in relatively lower flux via PP pathway as compared to SSK101. Lower flux via PPP would eventually result in correspondingly lower levels of intracellular NADPH due to which the cell would take longer to detoxify furfural from the media. On the other hand, the ability of SSK101 to metabolize glucose only via PPP would result in relatively higher NADPH load as compared to SSK42 and lead to quicker removal of furfural from the media. When glucose was substituted by xylose as the sole carbon source, neither SSK42 nor SSK101 could completely remove furfural from the medium even by 96 h (Fig. 4a). This observation can be explained with mechanism of xylose metabolism. Xylose enters central carbon metabolism via non-oxidative phase of PPP and results in bypass of 2 mol of NADPH formed in the oxidative phase as compared to glucose on a molar basis (Fig. 1). We predict that the scarcity of NADPH in the microbial cell is the reason for relatively lower rate of furfural removal from the medium when xylose is the sole carbon source.

#### Effect of osmotic stress

The hydrolysate generated by dilute acid treatment of lignocellulose results in the formation of a soluble fraction which contains pentose(mainly xylose) with minor component of hexose sugar (glucose). We thus chose a mixed carbon source consisting of glucose (minor component with 1% concentration) and xylose (major component with 4% concentration) to study the influence of osmotic stress. Glucose acts as an osmotic stressor at concentration > 100 mM (around 1.8%) while xylose exerts its stress at concentration > 120 mM (around 1.8%) under similar microaerobic conditions [28]. Thus, in our experimental setup, xylose at 4% is the main osmotic stressor and not the glucose which is at 1%. When the cells were grown in this medium in presence of 1 g/L furfural, strain SSK101 was able to clear furfural from AM1 medium within 24 h while SSK42 took additional 48 h for disappearance of furfural from the medium (Fig. 4b). However, at inhibitor concentration of 1.6 g/L, only marginal metabolism of furfural was observed for both SSK101 and SSK42 till 96 h; and medium containing SSK101 had relatively lower furfural as compared to the one containing SSK42.

*Escherichia coli* responds to osmotic stress by increasing intracellular glutathione levels [29] and inducing genes *soxS*, *sodA* and *katE* [30] that are also involved in combating oxidative stress. We must emphasize that osmotic stress is an additional element to the oxidative stress that is induced by furfural [15] itself. Under oxidative stress, glutathione is required to maintain reduced intracellular environment of the cell and in the process gets oxidized and needs to be reduced again. This regeneration of reduced glutathione is accomplished by oxidation of NADPH. We thus predict that osmotic stress introduces additional sink for the excess NADPH generated by strain SSK101.

#### Effect of media components

**Fig. 5:**
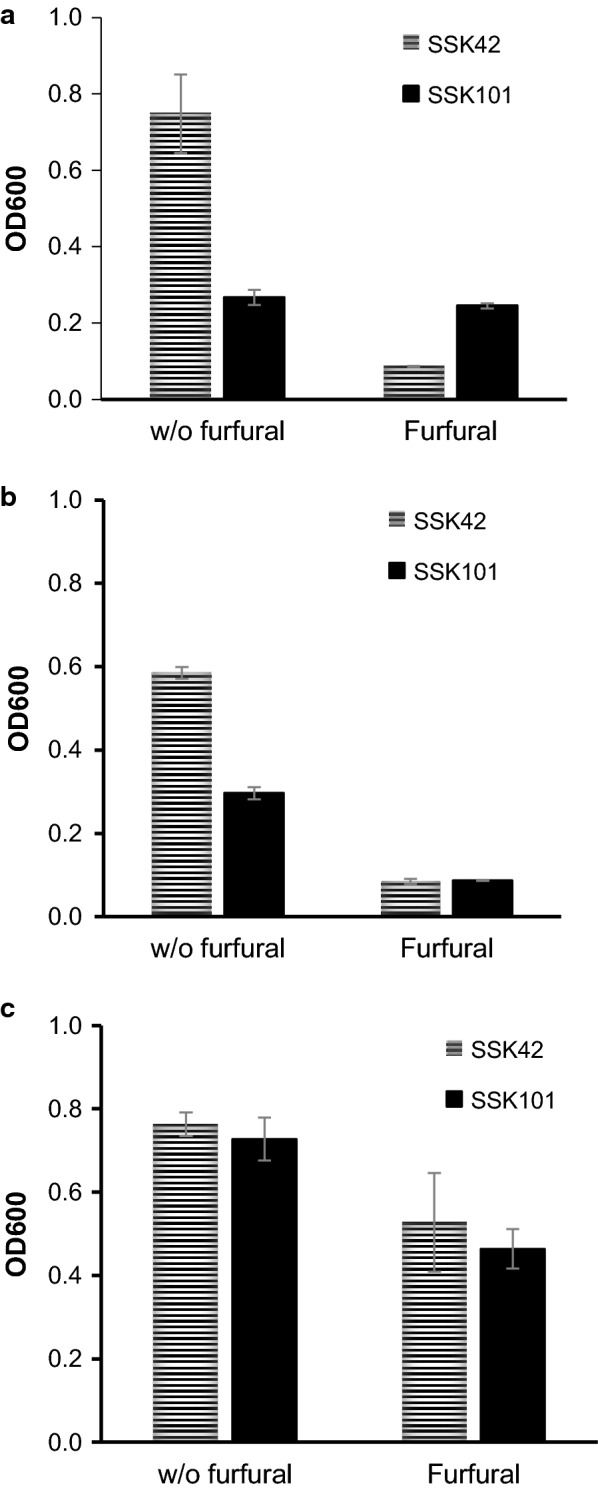
Role of media components in tolerance against furfural. Strains were cultured in Hungate tubes containing either AM1 medium with 1% glucose (**a**), 1% xylose (**b**), or in LB medium (**c**) and OD_600_ recorded at 24 h

We next tested the influence of media components upon furfural tolerance. AM1 is a minimal medium with high cellular biosynthetic requirements. Any perturbation of cellular homeostasis in minimal medium will elicit more pronounced physiological response as compared to that in a rich medium with low biosynthetic requirements. Our observation was along similar lines. When SSK42 was grown in AM1 medium containing either glucose (Fig. 5a) or xylose (Fig. 5b), we found 8.7-fold and 6.9-fold reduction in growth, respectively, in presence of furfural. Compared to SSK42, less growth was observed in SSK101 in AM1 medium. However, we did not observe any decline in growth due to furfural addition in case of SSK101 grown in AM1 + glucose (Fig. 5a), though growth did decline significantly when xylose was present in the AM1 medium (Fig. 5b). This clearly suggested role of excess NADPH in detoxifying furfural when SSK101 was grown in glucose medium. Interestingly, rich LB medium remarkably nullified the growth inhibition of SSK101 (Fig. 5c) that was observed for both AM1 + glucose (Fig. 5a) as well as AM1 + xylose (Fig. 5b) media in absence of furfural. This behavior could be attributed to both absence of glucose that would result in lower NADPH stress and lowered biosynthetic requirements of the cell in LB medium. Rich LB medium also, expectedly, buffered the toxicity of furfural as compared to that in AM1 medium, and growth of either strain resulted in relatively higher biomass (Fig. 5c). These results point to the fact that competitive advantage conferred on SSK101 strain by deletion of *pgi* gene is neutralized by rich complex media.

### Physiological evaluation at bioreactor level

Our results indicate that both strains can increase biomass in presence of either furfural or 5-HMF (Fig. 2), and increased osmotic stress also did not hinder them to metabolize 1 g/L furfural under the tested condition in Hungate tube (Fig. 4a). We next evaluated the physiological response of SSK101 and SSK42 under controlled conditions using a bioreactor. We increased complexity of investigation by including both furfural and 5-HMF at 1 g/L each leading to total inhibitor load of 2 g/L in bioreactor. This particular investigation assumes more importance since an earlier study has highlighted the synergistic inhibitory effect exerted by furfural in presence of 5-HMF on microbial metabolism [9]. We chose the concentration of respective furan in tune with the reported concentration of same in acid treated rice straw hydrolysate [6]. We also varied the concentration of sugars in the culture media to observe whether the concentration of glucose plays any role in microbial productivity. To prevent confounding effect due to variation in the osmotic load we compensated the change in glucose concentration by varying concentration of xylose. In all bioreactors, total sugar load was ~ 5.5%. It has been reported that in case of acidic treatment of hydrolysate and depending upon the severity of pretreatment the concentration of xylose to glucose exceeds in the range of 2–26 fold [31, 32]. In our studies the fold increase of xylose over glucose concentration was 4–8.3 fold.

**Fig. 6:**
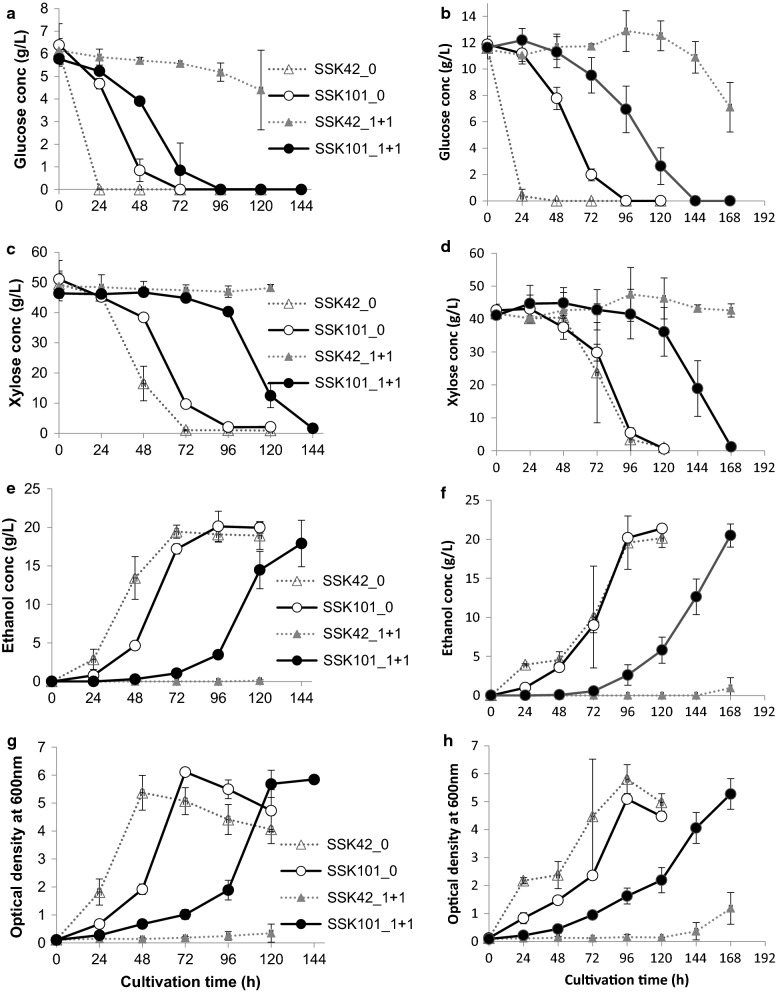
Influence of glucose concentration and binary stress of furfural and 5-HMF on fermentation of SSK101 and SSK42 in bioreactor. Strains were grown either at 6 g/L (**a**, **c**, **e**, **g**) or at 12 g/L (**b**, **d**, **f**, **h**) glucose concentration. (1 + 1) represents concentration of (furfural + 5-HMF) in g/L. 0 represents absence of inhibitors. Results represent mean of two independent replicates and error represents SD of mean

We observed very distinct behavior of SSK42 and SSK101 in the bioreactor when grown either in absence or in presence of the inhibitor mix. In the absence of inhibitors, both SSK42 and SSK101 were able to utilize the sugar mixture in about 96 h for both 6 g/L (Fig. 6a, c) as well as 12 g/L glucose (Fig. 6b, d). Expectedly, the rate of glucose utilization was faster in SSK42. The maximum ethanol titers for both strains at either concentration of glucose were also comparable (Table 1). At lower glucose concentration, SSK101 exhibited highest ethanol productivity at 48–72 h time interval, which shifted to later interval of 72–96 h at higher glucose concentration (Table 2). Similar pattern of improved ethanol productivity at lower glucose concentration was also observed for SSK42, which displayed highest ethanol productivity during 24–48 h at lower glucose concentration and during 72–96 h at higher glucose concentration (Table 2). In case of SSK42 we observed the classic diauxic growth pattern between 24 and 48 h (Fig. 6h) at higher glucose load which was also reflected in the pattern of ethanol production (Fig. 6f) at corresponding time points. One the other hand, SSK101 didn’t exhibit any diauxie at either glucose load. Interestingly, we didn’t observe any diauxic growth for SSK42 at lower glucose load (Fig. 6g). This likely explains the improvement in ethanol productivity for SSK42 (Table 2) at lower glucose concentration. Strain SSK101 co-utilized both glucose and xylose at tested concentrations of glucose.

**Table 1:** Influence of glucose concentration on sugar utilization and product synthesis in bioreactor

Strain	Maximum titer (g/L)			Product yield (g/g)			% Theoretical yield of ethanol	Rate of total sugar utilization (g/(L.h))	Rate of ethanol production (g/(L.h))
	Cells	Acetate	Ethanol	Cells	Acetate	Ethanol			
6 g/L glucose									
SSK42	2.69 ± 0.31	0.75 ± 0.01	19.44 ± 0.85	0.07 ± 0.01	0.01 ± 0.00	0.36 ± 0.05	69.94 ± 8.85	0.76 ± 0.06	0.27 ± 0.01
SSK101	3.06 ± 0.02	0.67 ± 0.08	20.13 ± 1.98	0.07 ± 0.01	0.01 ± 0.00	0.37 ± 0.08	71.80 ± 15.73	0.58 ± 0.07	0.21 ± 0.02
SSK101 + inhibitors	2.92 ± 0.06	1.17 ± 0.14	17.90 ± 3.02	0.06 ± 0.00	0.02 ± 0.00	0.35 ± 0.05	68.89 ± 9.26	0.35 ± 0.01	0.12 ± 0.02
12 g/L glucose									
SSK42	2.90 ± 0.26	1.41 ± 0.04	20.14 ± 1.20	0.06 ± 0.02	0.03 ± 0.00	0.39 ± 0.01	75.16 ± 1.33	0.43 ± 0.02	0.17 ± 0.01
SSK101	2.55 ± 0.07	0.97 ± 0.03	21.37 ± 0.22	0.05 ± 0.00	0.02 ± 0.00	0.40 ± 0.01	77.29 ± 2.23	0.45 ± 0.02	0.18 ± 0.00
SSK 101 + inhibitors	2.64 ± 0.27	1.16 ± 0.06	20.48 ± 1.47	0.05 ± 0.01	0.02 ± 0.00	0.40 ± 0.04	77.54 ± 7.05	0.31 ± 0.01	0.12 ± 0.01

Data for SSK42 with inhibitors (1 g/L Furfural + 1 g/L HMF) has not been given as no significant growth was observed here in either glucose concentration

**Table 2:** Influence of glucose concentration on ethanol productivity in bioreactor

Strain	Time interval (h) for maximum productivity	Maximum ethanol productivity (g/(L.h))
6 g/L glucose		
SSK42	24–48	0.44
SSK101	48–72	0.52
SSK101 + inhibitors	96–120	0.46
12 g/L glucose		
SSK42	72–96	0.40
SSK101	72–96	0.47
SSK101 + inhibitors	144–168	0.33

Data for SSK42 with inhibitors (1 g/L Furfural + 1 g/L HMF) has not been given as no significant growth was observed here in either glucose concentration

In the presence of the binary stress of furfural and 5-HMF, SSK101 completely utilized sugars under tested glucose regimes (Fig. 6a–d). In contrast, SSK42 that did not exhibit significant metabolic activity in either glucose concentration. As compared to cultivation without inhibitors, presence of inhibitors reduced productivity (Table 2) where highest ethanol titers were recorded after extended cultivation hours (Fig. 6e, f). Remarkably, even under inhibitor stress, maximum ethanol titers of SSK101 were comparable with that of SSK42 (and SSK101) observed in absence of inhibitors (Table 1). Maximum values of ethanol productivity of SSK101 under low glucose concentration was observed between 96 and 120 h and shifted to a later interval of 144–168 h at high glucose load (Table 2). At low and high glucose load the maximum ethanol yield of SSK101 was 69% and 78%, respectively. Although, these values are lower than those observed in lime treated hydrolysate where *E. coli* LY01 produced ethanol with > 90% of theoretical yield [7], the furfural and HMF concentration in this study were only 0.25 g/L and 0.34 g/L, respectively. Values of maximum ethanol yield at lower and higher glucose load was 0.35 and 0.40 g/g which were comparable with 0.39 value obtained by *E. coli* XW129 in nutrient and sodium metabisulfite supplemented hydrolysate [19]. SSK101 did not exhibit any diauxic growth pattern even in presence of inhibitors at either concentration of glucose tested. Though, SSK42 did not do well at total inhibitor load of 2 g/L, it did utilize both sugars and produced comparable ethanol titers at lower inhibitor load of 1 g/L with 0.5 g/L each of furfural and 5-HMF; and had an extended diauxie period (Additional file 1: Figure S1). Our efforts to further increase the stringency of cultivation conditions in bioreactors by increasing total sugar as well as furan load were not successful (Additional file 1: Figure S2).

Glucose overflow metabolism in *E. coli* is characterized by channelizing the glucose carbon to synthesis of NADH-independent acetate and is in response to accumulation of reduced nicotinamides in the cell [33]. We observed partial evidence of overflow metabolism in both SSK42 and SSK101 (Table 1). Both maximum titer of acetate and its difference between the two strains was reduced at lower concentration of glucose in absence of inhibitors. At lower glucose concentration, presence of inhibitors almost doubled the titers of acetate in SSK101 as compared to the control without any inhibitors. We believe that inhibitors place metabolic burden on the microbial cell with additional energy requirement and production of acetate is one way to fulfill increased ATP demand. Based on the results of bioreactor studies, we predict that the lower concentration of glucose reduces the NADPH stress on SSK101 while still being enough to channelize the energy and reducing equivalents required to balance anabolic reactions with detoxification of inhibitors.

## Conclusion

In this study, we developed an ethanologenic *E. coli* strain SSK101 by deletion of *pgi* gene of EMP-pathway in strain SSK42. Growth is promoted in strain SSK101 by both furfural and 5-hydroxymethyl furfural. Inhibitor tolerance of SSK101 is lost in absence of glucose. The tolerance advantage of SSK101 is neutralized in both complex LB media and in minimal AM1 media with xylose as sole carbon source. SSK101 can co-utilize both glucose and xylose in presence of furfural and 5-hydroxymethyl furfural without any diauxic growth pattern as observed in SSK42 at higher glucose loading. Glucose is utilized in initial fermentative stage to remove inhibitors from media while xylose is mainly utilized to increase biomass and produce ethanol. Results of this study have important implications in biofuel industry due to use of minimal media in investigations. In this study we report an ethanologenic *E. coli* strain SSK101 which can metabolize a mixture of sugars in minimal media in spite of synergistic inhibitory effect exerted by furfural in presence of 5-HMF.

## Methods

### Strains and media

*E. coli* strain SSK101 is a derivative of strain SSK42 which has undergone evolutionary adaptation [26] and has genotype as *E. coli* B P_gapA_PDH*ΔldhAΔfrdAΔpflB*. pCP20 was used to excise kanamycin cassette from SSK42 [34]. Phage based deletion of *pgi* gene was performed using KEIO mutant (JW3985-1) obtained from CGSC Yale collection. Replacement of *pgi* gene with kanamycin cassette in SSK42 was confirmed by PCR using primers pgiN_F_del (*CTCAACATTACGCTAACGGCAC*) and pgiN_R_del (*GCATCGACCTGTAGGCCTGATAA*). The *Δpgi* and kanamycin resistant strain thus generated was designated as SSK101. Genetic manipulation was performed in LB medium and physiological experiments to evaluate strain performance were performed in AM1 minimal medium with following composition—(NH_4_)_2_HPO_4_ (19.92 mM), NH_4_H_2_PO_4_ (7.56 mM), KCl (2 mM), MgSO_4_.7H_2_O (1.5 mM), Betaine HCl (1 mM), FeCl_3_.6H_2_O (8.88 µM), CoCl_2_.6H_2_O (1.26 µM), CuCl_2_.2H_2_O (0.88 µM), ZnCl_2_ (2.2 µM), Na_2_MoO_4_.2H_2_O (1.24 µM), H_3_BO_3_ (1.21 µM), MnCl_2_.4H_2_O (2.5 µM), CaCl_2_.2H_2_O (1.36 µM) [27]. Kanamycin was used at 12.5 mg/L concentration. All reagents used were molecular grade and obtained from either Sigma or HiMedia.

### Culture conditions

Shake flask experiment was carried out in 100 mL capacity flasks containing 25 mL AM1 medium. Hungate tube experiments were performed in capped glass tubes (18 mL capacity) containing 9 mL media and 1% of either glucose or xylose as sole carbon source as indicated against respective experiment. For shake flask experiment, primary culture was grown overnight in shake flasks containing AM1 medium with 1% glucose as sole carbon source. For Hungate tube experiments, primary culture was grown overnight in AM1 medium containing 1% xylose as carbon source. Primary culture for osmotic stress experiment contained a sugar mixture of 1% glucose + 4% xylose. In all cases, secondary culture was seeded at OD_600_ = 0.1. To limit gaseous exchange at multiple sampling time points, capped tube experiment was started in multiple tubes with each tube representing one time-point and respective tube was discarded after sampling. This allowed each tube to be uncapped just once at the required time-point only. Experiments were performed at 37 °C and at 150 RPM shaking, unless indicated. Each experiment was performed at least in biological duplicates.

### Fermenter conditions

Glycerol stock of respective strain was revived at 37 °C on AM1-agar plates containing 2% xylose as carbon source. Revived strain was streaked two more times on agar plate before inoculating in liquid AM1 medium containing 0.5% each of glucose and xylose in Hungate tube for 5 h at 37 °C in a gyrorotator. This culture was used to inoculate a primary fermenter vessel containing 200 mL AM1 medium with 0.5% each of glucose and xylose. pH was maintained at 7.0 using 2 N KOH and microaerobicity by pumping air in headspace at 0.03 LPM. After about 16 h of growth, the culture was used to inoculate secondary fermenter vessel at OD_600_ = 0.1. Carbon source in secondary fermenter was a combination of both glucose and xylose with concentration as mentioned in respective figures which represent results of at least two independent replicates. pH under all conditions was maintained at 7.0. Air flow in secondary fermenters during initial 24 h was 0.03 LPM and then increased to 0.04 LPM.

### Analytical methods

Change in biomass was measured by recording optical density at 600 nm using UV–Vis spectrophotometer (Ultrospec 3100—Amersham Biosciences). For Hungate tube experiments, furfural concentration was determined using UV–Vis method as reported before [35]. For fermenter studies, metabolites were estimated using Agilent HPLC with Aminex HPX 87H (300*7.8 mm) column and RI detector. Column temperature was maintained at 60 °C and that of detector at 50 °C. 4 mM H_2_SO_4_ was used as a mobile phase at flow rate of 0.6 mL/min. Metabolites were quantified using reference standard of 1 g/L obtained from Absolute Standards, USA.

R software (x64 ver.3.4.3) was used for t-test and generating heatmap. The heatmap in Fig. 2 was generated using the gplot package as follows. For making each sub-figure, the data was provided to the software in Excel sheet format. Depending upon the variation in values within the table, the software calculated and assigned the shade to each value of that particular table. For better clarity the color key for each subfigure (corresponding to its respective Excel sheet) has been separately denoted.

## Supplementary information

**Additional file 1: Table S1.** Rate of biomass formation in presence of furfural. **Table S2.** Rate of biomass formation in presence of 5-HMF. **Figure S1.** Influence of reduced inhibitor load on fermentation at 12 g/L glucose concentration in media containing glucose–xylose mixture. **Figure S2.** Fermentation profile of SSK101 at high sugar load (0.3% glucose + 9% xylose) and (A) 1.0 g/L each of both furfural and 5-HMF; (B) 1.5 g/L each of both furfural and 5-HMF.

## Data Availability

All data generated or analyzed during this study are included in this published article (and its Additional file 1).
