# Supplementary material for: Deletion of pgi gene in E. coli increases tolerance to furfural and 5-hydroxymethyl furfural in media containing glucose–xylose mixture
Source: Microb Cell Fact. 2020 Jul 28;19:153. doi: 10.1186/s12934-020-01414-0 (PMC7389444; doi:10.1186/s12934-020-01414-0)
Supplement: Supplementary file 1 — Additional file 1: Table S1. Rate of biomass formation in presence of furfural. Table S2. Rate of biomass formation in presence of 5-HMF. Figure S1. Influence of reduced inhibitor load on fermentation at 12 g/L glucose concentration in media containing glucose–xylose mixture. Figure S2. Fermentation profile of SSK101 at high sugar load (0.3% glucose + 9% xylose) and (A) 1.0 g/L each of both furfural and 5-HMF; (B) 1.5 g/L each of both furfural and 5-HMF. [file 12934_2020_1414_MOESM1_ESM.docx]

**Table S1: Rate of biomass formation in presence of furfural.** Cultures were grown in shake flasks containing AM1 media with 1% glucose as sole carbon source. Values are normalized to unit hour per sampling interval. The upper bold values represent SSK101 while lower values represent SSK42.

| Time interval | Furfural Concentration | | | | | |
| --- | --- | --- | --- | --- | --- | --- |
|  | 0 g/L | 1.0 g/L | 1.5g/L | 2.0 g/L | 2.5 g/L | 3.0 g/L |
| 0-3h | **0.014±0.005**  0.070±0.010 | **0.025±0.004**  0.013±0.004 | **0.016±0.003**  0.008±0.004 | **0.009±0.003**  0.005±0.003 | **0.005±0.001**  0.002±0.002 | **0.002±0.001**  0.001±0.002 |
| 3-6h | **0.029±0.003**  0.201±0.011 | **0.054±0.003**  0.044±0.005 | **0.028±0.002**  0.022±0.006 | **0.010±0.004**  0.011±0.005 | **0.003±0.003**  0.004±0.002 | **0.000±0.001**  0.004±0.006 |
| 6-9h | **0.047±0.009**  0.164±0.020 | **0.071±0.007**  0.107±0.024 | **0.062±0.015**  0.042±0.009 | **0.016±0.009**  0.010±0.008 | **0.005±0.002**  -0.001±0.003 | **-0.002±0.002**  -0.003±0.006 |
| 9-12h | **0.063±0.006**  0.007±0.013 | **-0.001±0.027** 0.167±0.009 | **0.074±0.009**  0.099±0.012 | **0.043±0.010**  0.038±0.011 | **0.008±0.009**  0.012±0.003 | **0.003±0.007**  0.002±0.003 |

**Table S2: Rate of biomass formation in presence of 5-HMF.** Cultures were grown in shake flasks containing AM1 media with 1% glucose as sole carbon source. Values are normalized to unit hour per sampling interval. The upper bold values represent SSK101 while lower values represent SSK42.

| Time duration | HMF Concentration | | | | | |
| --- | --- | --- | --- | --- | --- | --- |
|  | 0 g/L | 1.0 g/L | 1.5 g/L | 2.0 g/L | 2.5 g/L | 3.0 g/L |
| 0-3h | **0.011±0.005**  0.078±0.000 | **0.033±0.006**  0.041±0.001 | **0.030±0.005**  0.028±0.000 | **0.024±0.004**  0.020±0.000 | **0.018±0.003**  0.018±0.001 | **0.013±0.002**  0.014±0.001 |
| 3-6h | **0.027±0.002**  0.218±0.005 | **0.091±0.005**  0.146±0.005 | **0.096±0.004**  0.117±0.001 | **0.079±0.011**  0.090±0.001 | **0.052±0.013**  0.056±0.004 | **0.027±0.007**  0.033±0.002 |
| 6-9h | **0.044±0.006**  0.140±0.009 | **0.038±0.024**  0.158±0.012 | **0.091±0.039**  0.175±0.014 | **0.126±0.018**  0.124±0.011 | **0.131±0.010**  0.113±0.008 | **0.097±0.025**  0.076±0.001 |
| 9-12h | **0.067±0.012**  0.042±0.015 | **0.036±0.033**  0.063±0.037 | **-0.043±0.040**  0.079±0.013 | **0.002±0.023**  0.137±0.007 | **0.064±0.003**  0.138±0.011 | **0.095±0.015**  0.107±0.005 |

**
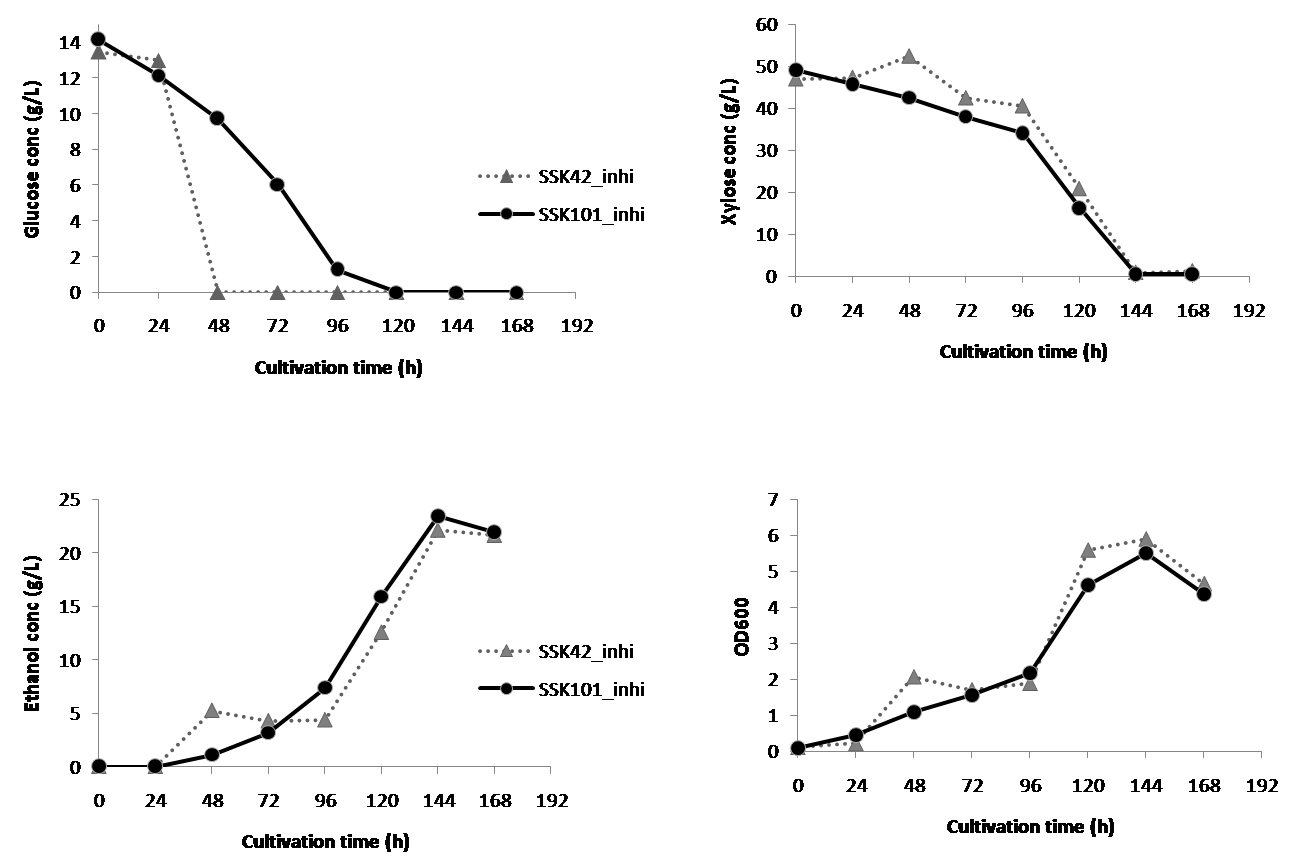
**

**Figure S1: Influence of reduced inhibitor load on fermentation at 12 g/L glucose concentration in media containing glucose-xylose mixture.** Strains were cultured in a bioreactor in presence of both furfural and 5-HMF with concentration of each at 0.5 g/L. Total sugar load was ~ 5.5%.


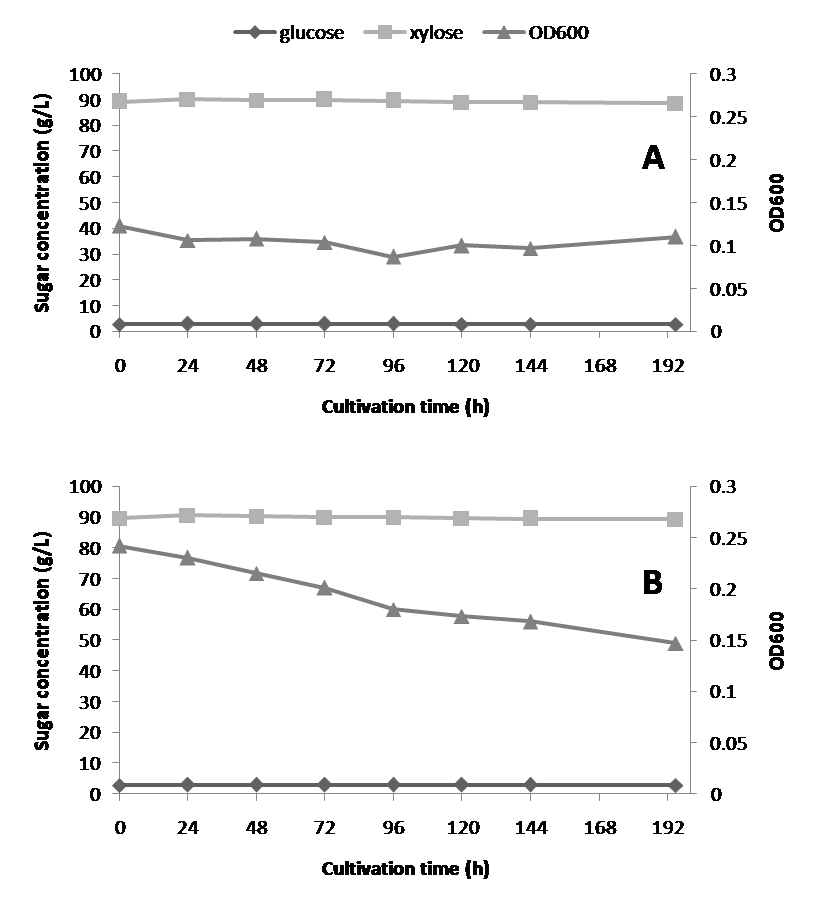


**Figure S2: Fermentation profile of SSK101 at high sugar mixture load (0.3% glucose + 9% xylose) and (A) 1.0 g/L each of both furfural and 5-HMF; (B) 1.5 g/L each of both furfural and 5-HMF.** Primary culture of SSK101 was performed in presence of 0.2% glucose + 0.8% xylose + 1 g/L furfural in the AM1 media.
